# Supplementary material for: Bayesian refinement of protein structures and ensembles against SAXS data using molecular dynamics
Source: PLoS Comput Biol. 2017 Oct 18;13(10):e1005800. doi: 10.1371/journal.pcbi.1005800 (PMC5662244; doi:10.1371/journal.pcbi.1005800)
Supplement: S1 Archive — (BZ2) [file pcbi.1005800.s011.tar.bz2 › gromacs_4.62_bayesian_saxs_refi/share/html/online/g_cluster.html]

g\_cluster


| g\_cluster Main Table of Contents |

**VERSION 4.6  
Sat 19 Jan 2013**

---

### Description

g\_cluster can cluster structures using several different methods.
Distances between structures can be determined from a trajectory
or read from an .xpm matrix file with the -dm option.
RMS deviation after fitting or RMS deviation of atom-pair distances
can be used to define the distance between structures.

single linkage: add a structure to a cluster when its distance to any
element of the cluster is less than cutoff.

Jarvis Patrick: add a structure to a cluster when this structure
and a structure in the cluster have each other as neighbors and
they have a least P neighbors in common. The neighbors
of a structure are the M closest structures or all structures within
cutoff.

Monte Carlo: reorder the RMSD matrix using Monte Carlo.

diagonalization: diagonalize the RMSD matrix.

gromos: use algorithm as described in Daura et al.
(Angew. Chem. Int. Ed. **1999**, 38, pp 236-240).
Count number of neighbors using cut-off, take structure with
largest number of neighbors with all its neighbors as cluster
and eliminate it from the pool of clusters. Repeat for remaining
structures in pool.

When the clustering algorithm assigns each structure to exactly one
cluster (single linkage, Jarvis Patrick and gromos) and a trajectory
file is supplied, the structure with
the smallest average distance to the others or the average structure
or all structures for each cluster will be written to a trajectory
file. When writing all structures, separate numbered files are made
for each cluster.

Two output files are always written:  
-o writes the RMSD values in the upper left half of the matrix
and a graphical depiction of the clusters in the lower right half
When -minstruct = 1 the graphical depiction is black
when two structures are in the same cluster.
When -minstruct > 1 different colors will be used for each
cluster.  
-g writes information on the options used and a detailed list
of all clusters and their members.

Additionally, a number of optional output files can be written:  
-dist writes the RMSD distribution.  
-ev writes the eigenvectors of the RMSD matrix
diagonalization.  
-sz writes the cluster sizes.  
-tr writes a matrix of the number transitions between
cluster pairs.  
-ntr writes the total number of transitions to or from
each cluster.  
-clid writes the cluster number as a function of time.  
-cl writes average (with option -av) or central
structure of each cluster or writes numbered files with cluster members
for a selected set of clusters (with option -wcl, depends on
-nst and -rmsmin). The center of a cluster is the
structure with the smallest average RMSD from all other structures
of the cluster.  

### Files

| option | filename | type | description |
| --- | --- | --- | --- |
| **-f** | traj.xtc | Input, Opt. | Trajectory: xtc trr trj gro g96 pdb cpt |
| **-s** | topol.tpr | Input, Opt. | Structure+mass(db): tpr tpb tpa gro g96 pdb |
| **-n** | index.ndx | Input, Opt. | Index file |
| **-dm** | rmsd.xpm | Input, Opt. | X PixMap compatible matrix file |
| **-o** | rmsd-clust.xpm | Output | X PixMap compatible matrix file |
| **-g** | cluster.log | Output | Log file |
| **-dist** | rmsd-dist.xvg | Output, Opt. | xvgr/xmgr file |
| **-ev** | rmsd-eig.xvg | Output, Opt. | xvgr/xmgr file |
| **-sz** | clust-size.xvg | Output, Opt. | xvgr/xmgr file |
| **-tr** | clust-trans.xpm | Output, Opt. | X PixMap compatible matrix file |
| **-ntr** | clust-trans.xvg | Output, Opt. | xvgr/xmgr file |
| **-clid** | clust-id.xvg | Output, Opt. | xvgr/xmgr file |
| **-cl** | clusters.pdb | Output, Opt. | Trajectory: xtc trr trj gro g96 pdb cpt |

### Other options

| option | type | default | description |
| --- | --- | --- | --- |
| **-[no]h** | bool | no | Print help info and quit || **-[no]version** | bool | no | Print version info and quit || **-nice** | int | 19 | Set the nicelevel || **-b** | time | 0 | First frame (ps) to read from trajectory || **-e** | time | 0 | Last frame (ps) to read from trajectory || **-dt** | time | 0 | Only use frame when t MOD dt = first time (ps) || **-tu** | enum | ps | Time unit: fs, ps, ns, us, ms or s || **-[no]w** | bool | no | View output .xvg, .xpm, .eps and .pdb files || **-xvg** | enum | xmgrace | xvg plot formatting: xmgrace, xmgr or none || **-[no]dista** | bool | no | Use RMSD of distances instead of RMS deviation || **-nlevels** | int | 40 | Discretize RMSD matrix in this number of levels || **-cutoff** | real | 0.1 | RMSD cut-off (nm) for two structures to be neighbor || **-[no]fit** | bool | yes | Use least squares fitting before RMSD calculation || **-max** | real | -1 | Maximum level in RMSD matrix || **-skip** | int | 1 | Only analyze every nr-th frame || **-[no]av** | bool | no | Write average iso middle structure for each cluster || **-wcl** | int | 0 | Write the structures for this number of clusters to numbered files || **-nst** | int | 1 | Only write all structures if more than this number of structures per cluster || **-rmsmin** | real | 0 | minimum rms difference with rest of cluster for writing structures || **-method** | enum | linkage | Method for cluster determination: linkage, jarvis-patrick, monte-carlo, diagonalization or gromos || **-minstruct** | int | 1 | Minimum number of structures in cluster for coloring in the .xpm file || **-[no]binary** | bool | no | Treat the RMSD matrix as consisting of 0 and 1, where the cut-off is given by -cutoff || **-M** | int | 10 | Number of nearest neighbors considered for Jarvis-Patrick algorithm, 0 is use cutoff || **-P** | int | 3 | Number of identical nearest neighbors required to form a cluster || **-seed** | int | 1993 | Random number seed for Monte Carlo clustering algorithm || **-niter** | int | 10000 | Number of iterations for MC || **-kT** | real | 0.001 | Boltzmann weighting factor for Monte Carlo optimization (zero turns off uphill steps) || **-[no]pbc** | bool | yes | PBC check |

---

http://www.gromacs.org  
gromacs@gromacs.org
